# The Conjoint Family Drawing: A Tool to Explore About Family Relationships

**DOI:** 10.3389/fpsyg.2022.884686

**Published:** 2022-06-13

**Authors:** Marialuisa Gennari, Giancarlo Tamanza

**Affiliations:** Department of Psychology, Catholic University of Sacred Heart, Milan, Italy

**Keywords:** family assessment, conjoint family drawing, assessment techniques, coding family drawing grid, types of family functioning

## Abstract

In this article we will present the Conjoint Family Drawing, a graphic-interactive tool developed to evaluate family relationships. This tool allows an analytical and clinical evaluation of families and their relationships while facilitating the understanding of the overall family functioning through a synthetic coding system which distinguishes families from each other. First of all, a presentation of the analytical coding system is provided; such system consists of a grid, formed by two distinct levels of observation: the analysis of the product, which, in turn focuses on two levels, the global-familiar one (given by the overall drawing) and the individual one (given the individual members’ drawings), and the analysis of the drawing process (what happens during the realization of the drawing), which is made up of the observations of family interactions at the individual and group level. Consistently with our objectives and the theoretical and methodological literature on family drawing in its various forms and ways of implementation, 10 indicators for product analysis and 9 indicators for process analysis have been identified. A sample of 117 Conjoint Family Drawings was analyzed in order to verify the coding system’s applicability and effectiveness. The sample was constituted according to a convenience (not probabilistic) criterion. Following, a computing system was developed to allow the investigation of the overall family functioning through three steps: (1) the analysis of the frequency distribution of each indicator, in order to verify the non-determinability rates and the distribution of the different answer options; (2) a two-step cluster analysis, to determine homogeneous groups of Conjoint Family Drawings and identify, within each cluster (and comparatively between the clusters), the indicators and answer modalities that mostly affect the clusters’ aggregation itself; (3) the development of a synthetic system to code the Conjoint Family Drawing, beginning with the indicators that define the typological profiles of the clusters obtained. The synthetic system was developed through a summative and logical-combinatorial method, merging the most discriminating and clinically significant coding items, that is, those that are best associable to specific ways of family functioning. Seven family types emerged from these analyses: families characterized by optimal functioning, families characterized by adequate functioning, families characterized by chaotic functioning, families characterized by fragile paternity, families characterized by separate functioning, families with multiproblematic functioning and residual families. The characteristics of these family types will be outlined in this article.

## Introduction: The Origins of the Conjoint Family Drawing

The drawing of human figures has been used in the field of psychological evaluation since the early years of the last century. Over time, it became widespread both for clinical assessment as well as for therapeutic purposes, as it is particularly suitable for children or people with speech impairments limiting their verbal communication skills (Goodenough, 1926).

The growing interest for drawings in the early decades of the past century led to the definition of a set of indicators capable of determining the correspondence between the subject’s cognitive development and the drawing’s characteristics. It was Machover (1949) who, in the early 50’s, suggested that human figure drawings could be used to gain an insight into the child’s inner world and, more generally, to facilitate the understanding of personality traits. The scholar showed how, thanks to identification and projection mechanisms, subjects used to transfer several elements concerning themselves onto their drawings. In this perspective, drawings go from being instruments for cognitive screening to becoming a key asset for the study of personality in a psychodynamic perspective. Therefore, they started being used with adults as well as with children. Starting from the 70’s on, Koppitz (1968, 1983) integrated the two above mentioned perspectives on the use of drawings and developed two subscales that allowed to measure both the subject’s cognitive development as well as his/her emotional condition (with particular reference to anxiety and conflict).

The Family Drawing Test landed in Europe in 1952 thanks to Porot (1952); at that time, both psychological research and clinical interventions focused on families. In particular, the idea of asking a young subject (6–15 years old) to draw his/her own family stems from the belief that families are the primary responsible for the individual emotional and cognitive development. The Family Drawing Test has been widely studied and refined by psychodynamically-oriented scholars and the many revisions provided over time have led to the development of several scales, each with specific indicators. More specifically, such indicators pertain the drawing’s content (Passi Tognazzo, 1975; Tambelli et al., 1996) on their side, focused on the shape when outlining their indicators.

The Kinetic Family Drawing (Burns and Kaufman, 1970; Burns, 1982; Handler and Habenicht, 1994) adds to the individual focus adopted by the Family Drawing Test by delving on the idea of the family as an active and interactive whole. The representation of families in action, of family members “doing something together,” allows to identify the child’s impressions of the relationships among the family members as well as to gain an insight of the active and creative nature of family dynamics. Burns (1982) specifically devised a category to assess family agency: beyond the formal characteristics considered by previous research, the author also developed some indicators to evaluate the various family activities depicted in the drawing (e.g., cooperation, communication, masochism, narcissism, sadism, tension, and support). The category named “style” includes indicators such as space fragmentation according to the number of subjects, encapsulation, presence of boundaries and dividing lines. Moreover, Handler and Habenicht (1994) proved that the Kinesic Family Drawing can correctly detect relational problems and it is a good measure of the style of family functioning.

Thanks to the development of the Collaborative Drawing Technique (Smith, 1985), interactional dynamics among family members took over and gained greater relevance over representational ones. This test gives the family—gathered around a table—an interactive, non-verbal task. To evaluate the final product, the authors developed both outcome (compliance to the instructions, individual involvement, scene depicted) as well as process indicators (use of the space, themes, and contents’ development).

The Conjoint Family Drawing we are presenting in this article is the result of the efforts of a group of researchers and clinicians from the Athenaeum Centre for Family Research and Studies of the Catholic University in Milan who have been working with divorced families over the past decades. This instrument has proven to be particularly effective in both clinical assessment and therapy and it has been used with different family types. Over the years, a coding grid for the analysis and interpretation of drawings has been developed on the basis of both the most robust and consolidated indicators found in the literature on family drawings and the most relevant and updated theoretical and clinical knowledge on family dynamics (Gennari and Tamanza, 2012).

## The Conjoint Family Drawing

The instrument administration requires all family members to be present together, each is called to pick a colored marker that will be used throughout the whole session so to distinguish the individual contributions of each family member. Family members are given the following instructions: “Now I would like you to use this sheet to draw your family while doing something. You can take some time to decide together what you would like to draw.”

Instructions are purposefully ambiguous and generic so to allow family members to take a shared decision on what to draw: the family has, therefore, a great freedom of decision and expression, the only limitation being the color of the marker chosen by each member. The instructions’ ambiguity serve the purpose of allowing family characteristics to emerge and family members to reveal their peculiar way of interacting with one another and of managing a common task.

Lastly, instructions “speak” to the family as a whole, considering it as a group whose members share a common history and have reciprocal relationships. In other words, the observation of the ways in which family members face a conjoint task (i.e., a drawing in our case) can provide an insight on the family’s characteristics and offer a glimpse of the specific and unique ways family members interact with one another.

## The Drawing’s Coding Grid

Consistently with the aims of the instrument and with the literature on both family and individual drawing, a coding grid to interpret the Conjoint Family Drawing was developed over the years. Such grid includes some useful indicators to evaluate the family functioning and its overall dynamics: the Gestalt perspective (Lobb, 2001), Olson’s circumflex model (Olson et al., 1979), family relations assessment instruments, such as the Family Life Space-FLS (Gozzoli and Tamanza, 1998) have all been used to develop such indicators. Other indicators assess couple’s dynamics, both in terms of the marital couple as well as the parental couple (Cigoli and Scabini, 2006); with respect to these latter indicators, the authors drew upon Minuchin’s (1974) strategic and structural therapy, the contextual therapy of Boszormenyi-Nagy and Spark (1973) and Bowen (1978) approach. Lastly, some indicators pertain to individual functioning and evaluate the individual either in relation to other family members or with regards to his/her specific functioning (these latter indicators are based on the literature on individual drawings, see, for example Burns, 1982; Handler and Habenicht, 1994; Fury et al., 1997; Jordan, 2001).

The coding grid is structured around four main areas of investigation: the overall family outcome, the individual outcomes produced by each member and the process at both the individual and family level.

The analysis of the **outcome** consists of an attentive and systematic observation of the drawing and does not require any other additional information besides the drawing itself. It is based upon four indicators:

1. *Occupation of the space*. Ideally, one should expect a balance between the space occupied by the drawing and the blank space (the drawing’s background), this latter symbolizing the possibility for change and transformation. On the contrary, the absence of blank space (filled up drawings) suggests the absence of a mental space to welcome the unexpected and might indicate that the family is stuck or caught up in painful or difficult circumstances. Conversely, a drawing full of blank spaces indicates the poverty and inability of the family members to provide an articulate and specific representation of themselves. It also suggests the lack of resources to face both expected and unexpected (in this case the drawing) challenges.
2. *Drawing’s realism*. This indicator assesses the realism of the family drawing. Is the drawing a photographic-like representation of the family or is the family not recognizable? Either way, symbolic or extremely confused drawings indicate a failure in complying to the instructions and a resistance, on the side of the members, to represent themselves as a family.
3. *Overall drawing quality*. This indicator has to do with the overall feeling emerging from the drawing: does it convey a positive, vital feeling or is it mournful and dark? In order to evaluate the drawing, both the content and realization modes (shapes, colors, objects, etc.) are considered. This indicator allows the researcher to understand whether the family is conceived as a nurturing and supportive environment by its members or if they perceive it as a place of sorrow and pain.
4. *Topic(s) represented*. The goal, in this case, is to determine whether the family is capable of depicting a common scene, as required by the instructions. Such an indicator, therefore, is particularly useful in highlighting the family’s capability of acting as a whole. In case the drawing lacks the presence of a unique scene involving the whole family, it is useful to analyze each of the family scenes depicted. Specifically, it is important to determine which subsystems take part in the creation of what scenes in order to understand which members work together toward a common goal. The various scenes could be the result of individual work or, rather, the outcome of a joint effort made by the couple members, the siblings or by intergenerational dyads, such as that composed by a parent and his/her child.

A further set of outcome indicators pertains the individual contributions given by each member. The observation of individual contributions allows to detect family coalitions, relational conflicts, and power struggles as well as the cooperative or exclusion dynamics and the reciprocal roles played by family members. To this end, the following indicators have been identified:

5. *Integration and participation in the drawing*. The goal of such indicator is to determine whether more people took part in the representation of one specific object in the drawing. People can participate by adding something and building on what was previously drawn or, at the contrary, by destroying, erasing or diminishing previous contributions. This indicator measures both the quantity and quality of the total interactions and of those within the parental couple.
6. *Family members represented in the drawing*. It is key to determine whether all family members appear in the drawing and who’s drawn them, similarly, it is important to notice if one member appears more than once as this is an important indicator of the family’s ability to share a common and recognizable representation of each member. In this perspective, the repeated presence of the same character is problematic as it indicates the family inability of acknowledging the contribution given by other members. In a similar fashion, the absence of a family member is an indicator of his/her perceived value and role within the family.
7. *Realism of the subjects represented*. Such indicator detects the “humanization” of the subjects represented in the drawing by determining whether they are consistent with the reality or, on the contrary, if they assume ambiguous or unreal shapes (e.g., symbols or inanimate objects). In other words, this indicator reveals the capability, on the side of the family members, to recognize each other as human beings.
8. *Characterization of the subjects.* This indicator pinpoints the details characterizing the human figures depicted in the drawing. According to the literature on human figure drawings, the presence or absence of details and the overall richness of a representation is an indicator of the relational and individual value of the person being represented.
9. *Presence of symbols.* In the case of Conjoint Family Drawings, symbols are defined as those graphical elements that, due to their unrelatedness to the overall scene, require a specific attention and interpretation on the observer’s side. With respect to the drawing evaluation, each symbol needs to be taken into account and its underlying meaning clinically understood.
10. *Presence of deletions.* The presence of deletions carried out by one family member to the detriment of another member’s graphic production can highlight conflicts and power struggles within the family; moreover, it is important to record who is performing the deletion and what is deleted, as the object being erased might be an indicator of the subject of the conflict.

The second level of analysis pertains to the observation of the drawing **process**. It necessarily implies the presence of a silent observer that codes the behaviors and attitudes of each family member throughout the whole session. Such an observation is therefore possible only provided that the researcher is present during the task execution or if a video-recording is taken.

As happened for outcome indicators, process indicators focus on both the overall family functioning as well as on the behaviors and individual contributions of each family member. Actually, being it extremely hard to distinguish the individual and family contributions as they are largely intertwined, most of the indicators involve two levels of observation: the detailed observation of each individual member and, subsequently, an overall impression of the family as a unity. Such an impression can be the result of the sum of individual actions or rather stem from a particularly relevant contribution by one member.

Following are the process analysis indicators:

11. *Involvement in the task.* Who accommodates the researcher’s request and tries to facilitate the involvement and compliance of the other family members? Determining who, among the members, is complying with the instructions is important to understand the level of motivation and alliance with the research/clinical team. Moreover, it indicates the presence of leading and organizing functions within the family. In particular, one can expect an initial involvement of the parental couple (either individually or as a couple), of the siblings (again, individually or as a group), or of a parent and a child together.
12. *Decision made*. Such an indicator focuses on the time allocated to the decision process and it particularly evaluates whether family members are capable of devoting a congruous amount of time to deciding what to draw. The lack of dialogue (immediate acting out) as well as a prolonged discussion signal an inability in effectively manage the decision process and might be indicators of a difficulty in coping with unforeseen situations or problems.
13. *Decision making modality*. With regards the decision-making process, it is important to acknowledge the nature of the exchanges between the family members as they can be more or less constructive, depending on whether they are capable of listening to each other and exchanging opinions in a fair and calm manner. In this sense, hostile or conflicting tones, the exclusion of one (or more) member(s), the refusal to take part in the decision-making process, the impossibility of reaching a shared decision, or the making of a one-sided choice are all signs of dysfunctional relational and communication dynamics.
14. *Emotional climate*. Focusing on the emotional climate of the family system throughout the drawing process allows to assess the family’s ability to cope with and find a shared solution to potential problems or stress factors (in this specific case the task of making a drawing). Specifically, this indicator aims to detect defense mechanisms, such as banalization and irony as well as to highlight the presence of negative emotional states, such as excessive anxiety or anguish. Moreover, this indicator signals the family’s ability to maintain a sufficient level of emotional organization to effectively carry out the task. Besides the evaluation of the role played by each family member, it is also important to assess the family as a whole (synthetic evaluation).
15. *Family exchanges during the drawing execution*. Such an indicator allows researchers to observe the family while moving around the sheet: do family members allow themselves to freely move around the sheet in order to realize the best possible shared outcome? The presupposition underlying such indicator is that the members’ immobilism might signal rigidity, lack of flexibility and adaptability. Similarly, excessive or haphazard movements might indicate the lack of defined roles and functions which result in a disorganization of the whole system. Along with the positions and actions taken by individual family members, it is also important to record the movements of the family as a whole (synthetic evaluation).
16. *Handling intergenerational difference*. When assessing the family, it is particularly important to examine the relationship between parents and their child(ren) while executing the task. With respect to parents, researchers should watch out for signs of containment (e.g., reminding the rules, setting and protecting boundaries, making proposals on how to carry on the task, etc.) and support (e.g., expressing sympathy and understanding, showing affection, encouraging and motivating, valuing the efforts made and the results obtained, etc.). The two parenting functions are named “father parenting” and “mother parenting.” With respect to children, it is important to determine whether they are capable of acknowledging and accepting their parents’ suggestions and support and therefore if they recognize their role and function within the family (called “acknowledgment of mother” and “acknowledgment of father”).
17. *Handling the intergenerational difference: siblings*. In this case, attention is placed on horizontal family ties, focusing on the sibling relationship and on the couple relationship alternatively. With respect to siblings, it is important to understand whether they are capable of differentiating from one another, each giving their own specific contribution to the common project, avoiding both homologation and forced differentiation.
18. *Handling the intergenerational difference: the marital couple*. With respect to spouses, the presence of a reciprocal acknowledgment of one’s role (e.g., valuing and referring to the other as a parent, supporting each other in parenting, promoting cooperative actions, etc.) as well as of mutual support (e.g., showing mutual support, sharing tasks, etc.) is investigated.

## The Drawings’ Analytic Evaluation

The categorical analysis of the Conjoint Family Drawing carried out through the coding grid provides a lot of useful information that may result in a deeper understanding of both individual and family functioning. The drawing allows an insight on the cognitive and developmental functioning of each member as well as on his/her characteristics, moreover it provides information on the interactional and relational dynamics within the family. It seems important to remind that, in order to maintain a multi-perspective and foster a multidimensional approach to the study of families, the individual and family levels of analysis need to be conceived as deeply intertwined. Another relevant aspect concerns the fact that there are three possible levels of observation that, while being strictly connected, pertain to different and complimentary areas: the representational level (that is the drawing itself), the operative level (that is the process of making the drawing), and the organizational level (how the family faces the task). By analyzing the actions undertaken to complete the drawing task, it is therefore possible to understand the family coping mechanisms.

In light of the above-mentioned characteristics, the drawing proves to be an extremely useful instrument for assessment, evaluation, and research purposes.

Moreover, the Conjoint Family Drawing can also be used in clinical and therapeutic settings as it increases the understanding of family dynamics while promoting change. Speculating over the drawing allows the family to retrieve the topics and themes emerged during the task execution while the contemplative and self-observational nature of the clinical context foster reflection and introspection. Moreover, the conjoint family experience, the act of “making something together,” is inherently transformative; it is by acting that the family acquires awareness of itself and its own patterns.

Lastly, the Conjoint Family Drawing can be used with different family types, being it suitable for families facing different phases of their life cycle (small children, adolescents, young adults) or dealing with difficult transitions (marital crises, traumatic events, such as illnesses or psychological symptoms). Moreover, this instrument can be used with families belonging to a different cultural background than that of the researchers and with families having limited language and cultural proficiency (e.g., multiproblematic families or low educated ones). The primordial linguistic code embedded in the gesture of making a drawing effectively contains the biases caused by lack of acculturation or poor education.

The Conjoint Family Drawing coding grid has an extremely significant heuristic component as it organizes all the relevant information regarding the family and its members in a systematic and controlled fashion. This allows a preliminary, and yet complete, overview of all the available data while at the same time shielding the researchers from slipping into the perceptual and cognitive biases that typically characterize this phase of information retrieval and decoding (distortions, omissions, neglect, underestimation, etc.). Therefore, the grid is a very valuable instrument as it allows an intersubjective and objective interpretation of each drawing both in clinical and research settings.

## Method: The Drawings’ Typologic Evaluation

In order to test its validity and applicability, the analytic coding grid was tested on a sample of 117 family drawings. To this end, the frequency distribution of each indicator was computed in order to assess non-determinability rates and observe the distribution of the answers across the categories. This allowed to determine the applicability and discriminant validity of each indicator.

Subsequently, a synthetic measure of the Conjoint Family Drawing was also attempted for three main reasons. First, along with the analytic observation of the interplay among family members, it is important to provide an overall overview of a complex and multidimensional object such as the family; in this perspective, several indicators simultaneously contribute to determine the family functioning. Secondly, the use of a synthetic measure of the Conjoint Family Drawing would allow comparisons among different cases. Finally, also in view of a multimethod approach to family relations, the use of a synthetic index might facilitate the integration and concomitant use of other measurement instruments. In order to achieve the aforementioned goals, we decided to:

(a) Use a clustering procedure (two-step cluster analysis, SPSS, 21) to group Conjoint Family Drawings into maximally homogeneous clusters and to identify, within each group as well as between the various groups, the indicators that had the greatest weight in determining the data aggregation and the clusters’ formation;
(b) Develop, starting from the prototypical family profiles emerging from the clusters, a synthetic-typological measurement system that could subsequently be associated to specific mechanisms of family functioning.

## Sample

The coding grid was empirically tested on a sample of 117 Conjoint Family Drawings in order to check its usefulness and test its efficacy. Sampling used a convenience approach. Table 1 shows the main characteristics of the sample.

**TABLE 1:** Sample characteristics.

Families characteristics	*%*
Non-clinical, Italian	27.4
Non-clinical, Immigrant	13.6
Clinical: Therapy	24.8
Clinical: Child custody evaluation	34.2
**Family composition**	**%**
1 Child	43.5
2 Children	47.9
3 Children	7.7
4 Children	0.9
**Age**	
Fathers	*M* = 41 (*SD* = 5.5)
Mothers	*M* = 38 (*SD* = 5.1)
Children	*M* = 6.3 (*SD* = 3)

With regards to education and job title, most of the families in our sample had a medium-high sociocultural background, this was especially true when the mothers’ education and job title was considered.

## Outcomes: Items Analysis

Two independent raters, external to the research team and having different theoretical backgrounds (i.e., cognitive and psychodynamic), coded the Conjoint Family Drawings after having received an appropriate training. Interrater agreement was very high (*K* = 0.998), thus proving the clarity and non-ambiguity of the indicators, the relative simplicity of the coding procedures as well as the handiness of the instrument itself. A second preliminary proof supporting the adequacy of the coding system was the fact that each of the drawings could be easily coded and that no indicator showed non-determinable values.

The univariate analysis of frequencies also showed that all indicators have a good discriminant validity. The two indicators that showed a slight polarization toward one answer can, therefore, be deemed acceptable (see Table 2).

**TABLE 2:** Indicators showing the most polarized responses.

*7*. Realism of the subjects being represented					
	**Consistent with reality**	**Ambiguous**		**Inadequate**	**n.d.**
	72.6	19.7		4.3	0.0
*16 Parental functions*					
	**Present**	**Uncertain**	**Absent**	**Excessive**	**n.d.**
*16f. Acknowledgment of mother*	73.5	24.8	1.7	0.0	0.0

## Outcomes: Cluster Analysis

The cluster analysis resulted in a two-cluster solution, each showing rather robust cohesion and separation coefficients (0.3). The two clusters, including 113 of the 117 drawings in the sample, show a good internal homogeneity and are significantly different from one another. Specifically, one cluster is characterized by a high internal homogeneity and includes clearly dysfunctional values with respect to all the indicators considered, the other cluster is more heterogeneous as it includes both functional as well as intermediate values but no dysfunctional value.

Since the outcomes of the cluster analysis also depend upon the number of indicators and their homogeneity, that is by their belonging to the same theoretical domain, we decided to run two independent cluster analyses, separating the process and the outcome indicators. Such additional level of analysis allowed the researchers to determine whether clearer and qualitatively more significant profiles could emerge from the sample. The cluster analysis on the outcome indicators resulted in four clusters, with an acceptable cohesion and separation coefficient (0.2). The four clusters almost cover the entire sample (113 cases out of 117).

The indicators having the greatest weight in determining cluster membership were: integrations’ quantity (with a value of 1), integrations’ quality (0.805), drawing’s realism (0.703), topics represented (0.577), overall quality of the representation (0.547), and space occupation (0.531). On the contrary, the indicators having the least importance in determining the clusters were: family members represented in the drawing (0.267), subjects’ realism (0.252), characterization of the subjects (0.236), couple’s integration quality (0.235), presence of symbols or deletions (0.059).

Only the six indicators that scored above the cut-off threshold and that, therefore, had a significant role in determining the clusters aggregation were taken into account and their distribution within each cluster was closely examined in order to understand the specificities characterizing each cluster and to provide a clinical interpretation (see Table 3).

**TABLE 3:** Cluster analysis on the outcome indicators.

Cluster	Characteristics of the cluster
*1^°^ cluster (39.8% of the sample)-Adequate drawings*	Indicators are positive and the overall representation is harmonic and collaborative.
*2^°^ cluster (29.2% of the sample)- Chaotic drawings*	Ambivalence; the drawings show the simultaneous presence of contrasting indicators: some are positive, others show problematic aspects.
*3^°^ cluster (19.5% of the sample)- Problematic drawings*	All the indicators considered clusters around the most problematic modalities.
*4^°^ cluster (11.5% of the sample)- Separated drawings*	Total lack of integration between the elements of the drawing, with a strong prevalence of individual elements. Fragmented and scarcely integrated drawings.

A two-step cluster analysis was also performed on the eight process indicators, that is, those pertaining the family interactions. Since we were planning to combine the two sets of indicators (i.e., the process and outcome indicators) in order to develop a synthetic and comprehensive assessment system of the Conjoint Family Drawing, we decided to perform a supervised clustering, setting the number of clusters to four.

The four clusters showed an acceptable silhouette coefficient of cohesion and separation (0.2). The most significant indicators in the formation of clusters were: handling of the difference within the marital couple (with a value of 1), decision making modality (0.844), father’s parenting (0.664), emotional climate (0.610), and family exchanges during the task execution (0.543). The remaining five indicators, namely, father’s acknowledgment (0.484), time allocated to the decision-making process (0.483), involvement in the task (0.424), mother’s parenting (0.216), and mother’s acknowledgment (0.214), showed below threshold values. All the 117 drawings were included in the so-formed clusters (see Table 4).

**TABLE 4:** Cluster analysis on the process indicators.

Cluster	Characteristics of the cluster
*1^°^ cluster (36.8% of the sample)- Cohesive family*	Positive interactions with respect to all family members. Well-functioning family in which members are capable of dialogue.
*2^°^ cluster (29.9% of the sample)- Problematic interactions within the family*	The decision-making process is hindered by conflicts, withdrawal, avoidance, or passive acceptance; poor family interactions and frequent attempts to belittle one another on the side of the spouses.
*3^°^ cluster (21.4% of the sample)- problematic parental guidance and positive family decision-making process*	Ambivalence within the parental couple, uncertainty of the father’s presence, presence of a serene emotional climate, static family exchanges, AND problems in the decision-making process.
*4^°^ cluster (12% of the sample)- problematic parental guidance but functional coping mechanisms*	Ambivalence within the parental couple, uncertainty of the father’s presence, presence of a serene emotional climate, static family exchanges BUT family members are capable of finding a shared solution and of using functional coping mechanisms

### The Drawings’ Synthetic Evaluation

If we take into consideration the six outcome indicators and the five process indicators that resulted as having the highest discriminant values (i.e., > di 0.5), we can immediately identify a set of answer modalities that give rise to two distinguishable and well-defined prototypical family profiles: the generative family configuration and the multiproblematic family configuration (see Table 5).

**TABLE 5:** Prototypical profiles.

	Generative	Multiproblematic
*Occupation of space*	Balanced	Poor, overfilled
*Overall quality*	Vital	Ambiguous or non-vital
*Topic(s) represented*	Family chooses one topic	Multiple topics: Individual actions or horizontal alliances between members
*Number of integrations*	Presences = absences	Absent or absences > presences
*Type of interaction*	Constructive/mainly constructive	Absent, mainly disruptive
*Drawing realism*	Congruent	Inadequate
*Decision-making*	Shared or conflict negotiation	Passive acceptance, avoidance or non-negotiated conflicts
*Emotional climate*	Acceptable levels of anxiety	Banalization or anguish
*Family exchange*	Dynamic, participated	Dishomogeneous, hyperkinetic or static
*Father function*	Present	Absent or overly present
*Support within the couple*	Valorization	Belittlement

The generative family configuration was further divided into two groupings, one corresponding to optimal family functioning and the other to good-enough functioning. In order to be classified as part of either grouping, a drawing should simultaneously satisfy at least 8 of the 11 indicators. The choice of setting the cut-off value to eight stems from the acknowledgment that only the presence of eight indicators could guarantee the prevalence criterion to be satisfied in both the overall indicators set as well as in each group of indicators (i.e., process and outcome) at the same time.

Therefore, the inclusion criteria for a case to be assigned to either configuration are:

– for a drawing to be included in the optimal-generative typology, all the 11 indicators need to assume functional and generative modalities (see Table 5);
– for a drawing to be included in the good-enough typology, there need to be from 8 to 10 generative prototypical indicators;
– if 8 or more indicators assume dysfunctional modalities, the drawing is to be attributed to the dysfunctional, multiproblematic configuration;
– all the other drawings should, at this point of the coding process, be classified as intermediate configurations.

By applying the aforementioned rules to our sample, the 117 Conjoint Family Drawings can be classified as follows in Table 6:

**TABLE 6:** Frequency distribution of the first family typologies emerged.

Family typologies	*N* ^°^	*%*
*Optimal-generative*	14	12.0
*Good-enough*	23	19.7
*Intermediate*	64	54.6
*Dysfunctional*	16	13.7
Total	117	100.0

In this perspective, we deemed it useful to focus on the intermediate configurations, using a qualitative logic. The previous application of quantitative criteria allowed the attribution of the drawings to a specific—intermediate—position, in between the generative and the problematic profiles. This specific positioning remains a common characteristic shared by all drawings in this subgroup, beyond further potential partitions. It seems important to remember that the two independent cluster analyses performed separately on the process and outcome indicators allowed to acknowledge the qualitative and clinical relevance of some of the descriptors and their capability of successfully discriminating among different family configurations. If we retrieve the key characteristics of the clusters emerged from the outcome and process analyses we can, therefore, conclude that:

(1) One of the clusters was characterized by the presence of contrasting variables that, while not completely hindering family action, rendered the image of a chaotic, disorganized functioning. With respect to our synthetic coding system, that is to the 11 outcome and process indicators, this implies a balanced presence of both generative and problematic indicators. This means that, for a drawing to be attributed to this typology, at least four generative and as many problematic variables should be simultaneously present.
(2) Ambiguity and belittlement within the couple along with an ambiguous or frankly problematic position on the side of fathers (that were either absent or overly present) characterized a second cluster. It should be reminded that family action could not be classified as solely problematic as family members also proved to be capable of producing integrated and collaborative drawings. Therefore, in order to be included in this family configuration, that we named as problematic parenting, the following inclusion criteria should be satisfied:
  – ambivalence or belittlement within the couple;
  – ambiguous, absent or excessively present fathering figures;
  – good levels of integration in the graphic production (presence of integrations ≥ absence of integrations).
(3) The third cluster was characterized by the lack of integration within the drawings, to the point that these family were referred to as separated or disengaged. Inclusion criteria require the simultaneous presence of the following:
  – complete lack of integration with the parental couple;
  – individual involvement in the task execution;
  – absence (or limited presence) of intergenerational integrations.

Table 7 shows the differentiation criteria for the drawings falling into the intermediate configurations.

**TABLE 7:** Differentiation criteria for the intermediate configurations.

Chaotic configuration	Balanced presence of generative and multiproblematic indicators
*Fragile parenting configuration*	Ambivalence and belittlement within the couple; ambiguous father figures: fathers are absent or overly present; good levels of integration within the graphic production (presences of integration= absences of integration).
*Separated or disengaged configuration*	Complete lack of integration within the couple; Individual commitment to the task; Absence (or limited presence) of intergenerational integrations.

In order to assess the appropriateness and functionality of such rules, they have been applied to the subsample of drawings falling into this intermediate category (see Table 7). The final goal was to determine whether they gave rise to mutually exclusive and well-differentiated groupings or, on the contrary, if the resulting groups were confused and overlapping. Results show an overlapping of only two cases out of 64 (3.1%). Such a result provides ground for the rules we set.

All the Conjoint Family Drawings falling in the so-called intermediate configuration could be reallocated to one of the three sub-groups (i.e., chaotic, fragile or separated/disengaged configuration) by using the synthetic computational system we developed. This constitutes an additional proof of its validity and suitability to measure family interactions. In this respect, the computing system seems to be particularly appropriate, both because the frequency distribution appears to be balanced, and because only the 12% of the cases in this category could not be reattributed to one of the subgroups.

**FIGURE 1:**
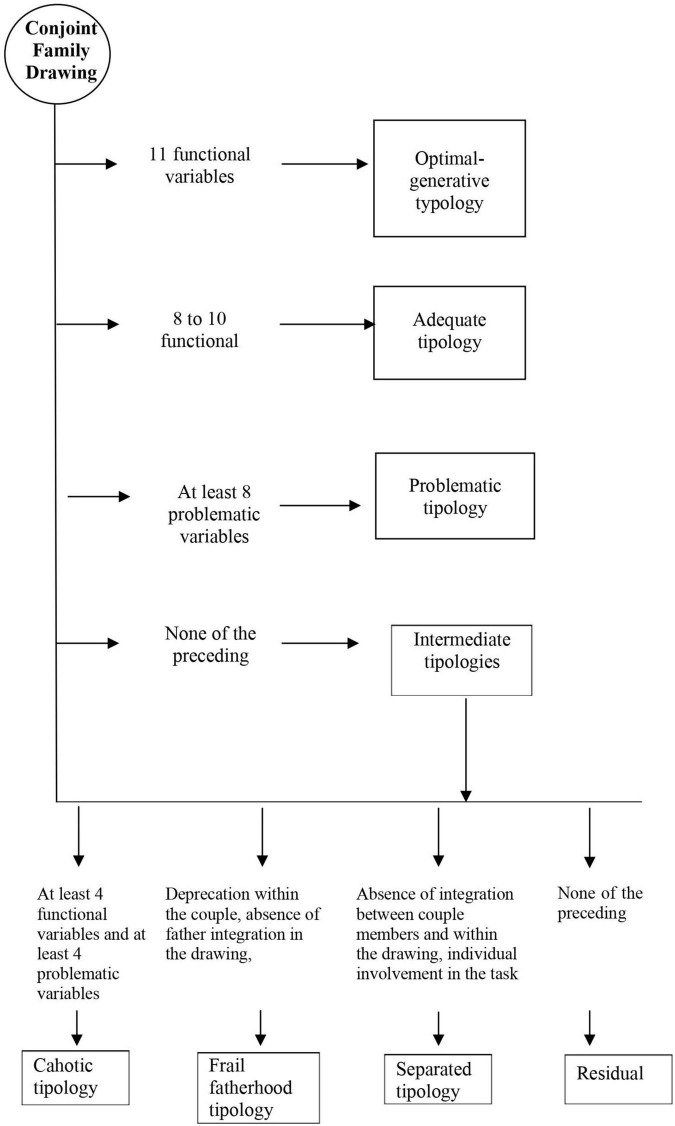
Decision tree for the identification of family relational configurations.

In short, the Conjoint Family Drawing synthetic evaluation system appears as a logic-conditional path (see Figure 1), in which a set of consecutive passages lead to each drawing being assigned to a given typology that, in turn, corresponds to a specific family relational configuration. Specifically, if a design cannot be attributed to the optimal, adequate and multi-problematic typological configurations, it is attributed to the intermediate configurations. In this case, the 11 discriminating indicators identified by the outcome and process clusters are analyzed, concluding that:

(a) If at least 4 functional indicators and at least 4 problematic indicators are found, a chaotic family functioning emerges;
(b) If deprecation within the couple, absence of father, integration between family members in the drawing are found at the same time, a “fragile paternity” family functioning emerges;
(c) If the absence of integration between couple members and within the drawing is accompanied by an individual involvement in the task, a separate family functioning emerges;
(d) If the drawing cannot be attributed to the optimal, adequate, nor to the multi-problematic typology and, at the same time, the conditions listed above (a-c) do not occur, one should conclude the drawing cannot be assigned to a specific typology; nonetheless, a clinical interpretation of the drawing can be provided by analyzing individual indicators (see paragraph 4 on the drawings’ analytic evaluation).

Finally, a further proof of the soundness of the synthetic evaluation system we developed was provided by associating the Family Configurations we observed with the characteristics of the various family groups constituting our sample (see Table 8).

**TABLE 8:** Bivariate analysis comparing family characteristics to typological Configurations.

	Italian %	Foreign %	Court mandated %	In treatment %
Generative/Optimal	18.2	12.5	15.0	0.0
Generative-good-enough	33.3	43.8	12.5	0.0
Multiproblematic	6.1	0.0	10.0	35.7
*Intermediate typology*				
Chaotic	3.0	25.0	15.0	10.7
Fragile parenting	15.2	0.0	30.0	14.3
Separated/Disengaged	15.2	0.0	7.5	25.0
Residual	9.0	18.7	10.0	14.3
Total	100.0	100.0	100.0	100.0

*Pearson χ^2^ = 51.758; df = 18; p = 0.000.*

The outcomes of this analysis clearly show a correspondence between the typologies obtained through the Conjoint Family Drawing synthetic evaluation system and the characteristics of the families in our sample. Specifically, more than half of the non-clinical families that were met in research contexts fell into the generative/optimal configuration while only a few were found to belong to the multiproblematic group. On the contrary, families that were recruited in clinical contexts were more likely to fall into the multiproblematic profile and never happened to be assigned to the generative group.

## Conclusion

The Conjoint Family Drawing provides an answer to the increasing need for tools for family assessment (Handler and Thomas, 2014; Pourhosein et al., 2016; Klumpp et al., 2020). It allows to read family functioning both in clinical (Pace et al., 2021) and socio-educational situations (Van Velsor and Cox, 2000), as well as in research settings. This tool might come in handy, especially in cases of speech or verbal impairments as it doesn’t draw upon the dialogic-narrative channel as a source of information; specifically, by leveraging on images, it can facilitate the access to the interactive and symbolic dimensions of family exchanges.

Thanks to its characteristics, the Conjoint Family Drawing is particularly suitable for the assessment of families with children aged between 3 and 13. One limitation of this instruments is that it is not suitable for families with adolescents or young adults, since drawing, at this stage of one’s development, is not an elective language and therefore young people may experience difficulties in using such mean of expression (Oster and Crone, 2004). Furthermore, assigning the family a drawing task may elicit counter-dependent behaviors in adolescents or constitute a regressive situation (Riley, 2001; Zeevi, 2021). Another limitation of this instruments is that, while being particularly suitable for the assessment and therapy of families in traditional settings, it cannot be used in on-line consultations, that are rapidly becoming extremely widespread (Kim et al., 2011).

The present article addresses the issue of the effectiveness of The Conjoint Family Drawing as no empirical evidence of its validity as a family assessment instrument has even been provided. Specifically, our study aims to fill this gap by empirically validating this instrument. To this end, a clear and well-defined coding grid was developed and the discriminant validity of the indicators was assessed. Our goal was to provide preliminary empirical evidence supporting the use of Conjoint Family Drawings in both research (Accordini et al., 2018; Mannino et al., 2019) and clinical settings (Gennari and Tamanza, 2017; Tamanza et al., 2018).

Further studies involving larger samples and specific target samples are needed in order to confirm the discriminant validity of this instrument (Pace et al., 2021). Moreover, studies comparing the outcomes obtained from the administration of the Conjoint Family Drawing with results from other assessment techniques are needed in order to prove the instrument’s reliability and specificity.

## Data Availability Statement

The raw data supporting the conclusions of this article will be made available by the authors, without undue reservation.

## Ethics Statement

The studies involving human participants were reviewed and approved by the Catholic University Ethics Committee. Written informed consent to participate in this study was provided by the participants’ legal guardian/next of kin.

## Author Contributions

MG and GT contributed to the conception and design of the study, performed the statistical analysis, and wrote all the sections of the manuscript. Both authors contributed to manuscript revision, read, and approved the submitted version.

## Conflict of Interest

The authors declare that the research was conducted in the absence of any commercial or financial relationships that could be construed as a potential conflict of interest.

## Publisher’s Note

All claims expressed in this article are solely those of the authors and do not necessarily represent those of their affiliated organizations, or those of the publisher, the editors and the reviewers. Any product that may be evaluated in this article, or claim that may be made by its manufacturer, is not guaranteed or endorsed by the publisher.
